# Grape Proanthocyanidins Induce Apoptosis by Loss of Mitochondrial Membrane Potential of Human Non-Small Cell Lung Cancer Cells *In Vitro* and *In Vivo*

**DOI:** 10.1371/journal.pone.0027444

**Published:** 2011-11-08

**Authors:** Tripti Singh, Som D. Sharma, Santosh K. Katiyar

**Affiliations:** 1 Birmingham Veterans Affairs Medical Center, Birmingham, Alabama, United States of America; 2 Department of Dermatology, University of Alabama at Birmingham, Birmingham, Alabama, United States of America; 3 Nutrition Obesity Research Center, University of Alabama at Birmingham, Birmingham, Alabama, United States of America; 4 Comprehensive Cancer Center, University of Alabama at Birmingham, Birmingham, Alabama, United States of America; University of Nebraska Medical Center, United States of America

## Abstract

Lung cancer remains the leading cause of cancer-related deaths worldwide, and non-small cell lung cancer (NSCLC) represents approximately 80% of total lung cancer cases. The use of non-toxic dietary phytochemicals can be considered as a chemotherapeutic strategy for the management of the NSCLC. Here, we report that grape seed proanthocyanidins (GSPs) induce apoptosis of NSCLC cells, A549 and H1299, *in vitro* which is mediated through increased expression of pro-apoptotic protein Bax, decreased expression of anti-apoptotic proteins Bcl2 and Bcl-xl, disruption of mitochondrial membrane potential, and activation of caspases 9, 3 and poly (ADP-ribose) polymerase (PARP). Pre-treatment of A549 and H1299 cells with the caspase-3 inhibitor (z-DEVD-fmk) significantly blocked the GSPs-induced apoptosis of these cells confirmed that GSPs-induced apoptosis is mediated through activation of caspases-3. Treatments of A549 and H1299 cells with GSPs resulted in an increase in G1 arrest. G0/G1 phase of the cell cycle is known to be controlled by cyclin dependent kinases (Cdk), cyclin-dependent kinase inhibitors (Cdki) and cyclins. Our western blot analyses showed that GSPs-induced G1 cell cycle arrest was mediated through the increased expression of Cdki proteins (Cip1/p21 and Kip1/p27), and a simultaneous decrease in the levels of Cdk2, Cdk4, Cdk6 and cyclins. Further, administration of 50, 100 or 200 mg GSPs/kg body weight of mice by oral gavage (5 d/week) markedly inhibited the growth of *s.c.* A549 and H1299 lung tumor xenografts in athymic nude mice, which was associated with the induction of apoptotic cell death, increased expression of Bax, reduced expression of anti-apoptotic proteins and activation of caspase-3 in tumor xenograft cells. Based on the data obtained in animal study, human equivalent dose of GSPs was calculated, which seems affordable and attainable. Together, these results suggest that GSPs may represent a potential therapeutic agent for the non-small cell lung cancer.

## Introduction

Lung cancer remains the leading cause of cancer related deaths in the United States and worldwide [1]. One of every three cancer-related deaths is attributable to lung cancer, and the dismal 5-year survival rate of approximately 14% has shown no improvement over the past three decades [2], [3]. Small-cell lung cancer and non-small-cell lung cancer (NSCLC) account for 90% of all lung cancers. NSCLC represents approximately 80% of all types of lung cancer and includes squamous cell carcinomas, adenocarcinomas, and large-cell carcinomas [4], [5]. Although a combination of chemotherapy and radiation therapy can improve survival of the patients, most patients die of disease progression, often resulting from acquired or intrinsic resistance to chemotherapeutic drugs [6]. Therefore, the exploration and development of more effective therapeutic agents and therapies that can target the molecules associated with tumor growth and apoptosis resistance will lead to improved outcomes in patients with lung cancer.

Natural plant products offer promising new options for the development of more effective chemotherapeutic strategies for cancers of various organs. Grape seed proanthocyanidins (GSPs) are promising phytochemicals that have anti-inflammatory [7] and anti-oxidant properties [8]–[10], and appear to exhibit minimal toxicity in laboratory animals [9], [10]. GSPs are readily extracted from grape-seeds, a by-product of grape juice and wine industries, and are a mixture of several polyphenolic components, which constitute dimers, trimers, tetramers, and oligomers/polymers of monomeric catechins and/or (-)-epicatechins, as described previously [9], [10]. We believe that at least some of the constituents present in GSPs act synergistically and may provide better chemotherapeutic effects than a single constituent.

Previously, we have shown that dietary supplementation of GSPs with AIN76A control diet resulted in a dose-dependent inhibition of the growth of A549 and H1299 NSCLC tumor xenograft in athymic nude mice, and the growth inhibitory effect of GSPs on the NSCLC xenograft tumors was associated with the enhancement of the levels of insulin-like growth factor binding protein-3 and anti-angiogenic effects in the tumor microenvironments (11). In another study, we also have reported that GSPs inhibit the proliferation and induce apoptosis of NSCLC cells *in vitro* and *in vivo* tumor xenografts, which was associated with their inhibitory effects on the cyclooxygenase-2 expression and production of its prostaglandin metabolite, PGE_2_ (12). In contrast, a significant inhibition of cell proliferation and induction of apoptosis in normal human bronchial epithelial cells after GSPs treatment under identical conditions was not observed [11], [12]. In spite of anti-carcinogenic effects of GSPs on NSCLC cells, a precise mechanism of the inhibitory effect on the NSCLC cell growth and apoptosis by GSPs is not well understood. In the present communication, we conducted a comprehensive investigation on the mechanism responsible for the inhibition of lung cancer cell proliferation and apoptosis using A549 and H1299 cell lines as an *in vitro* cell culture model and *in vivo* tumor xenograft model. To study the *in vivo* effect of GSPs on tumor xenograft growth, GSPs was given to mice by oral gavage 5 days/week. We report that GSPs-induced apoptotic cell death of NSCLC cells is mediated through modulations in the expression levels of pro- and anti-apoptotic proteins, loss of mitochondrial membrane potential and caspase-3 activation pathways. GSPs also checked the deregulated cell cycle progression and associated regulatory proteins in NSCLC cells. Thus our studies provide insight into the mechanism by which GSPs induce apoptosis in these cells. Additionally, our results provide a convincing rationale for the pharmacological activity of GSPs against human non-small cell lung cancer cells.

## Materials and Methods

### Reagents, chemicals and antibodies

The GSPs used in this study were obtained from Kikkoman Corporation (Noda, Japan). The JC-1 Mitochondrial Membrane Potential Detection Kit, and Anti-OxPhos Complex IV subunit IV (Cox IV) were purchased from Molecular Probes, Inc. (Eugene, OR). Cell Death Detection ELISA Kits were obtained from Roche Diagnostic Corporation (Indianapolis, IN). The primary antibodies were purchased as follows: antibodies for Bcl-2, Bcl-xl, Bax, caspase-9, cleaved caspase-9 and -3, anti-poly(ADP-ribose) polymerase (PARP) and β-actin were purchased from Cell Signaling Technology (Beverly, MA); antibodies for cytochrome c, Smac/DIABLO, Cyclin D1, Cyclin D2, Cyclin E, Cdk2, Cdk4, Cdk6, Cip1/p21, Kip1/p27 and the secondary antibodies, horseradish peroxidase-linked antimouse immunoglobulin G and anti-rabbit immunoglobulin G were obtained from Santa Cruz Biotechnology, Inc. (Santa Cruz, CA). Caspase-3-specific inhibitor (z-DEVD-fmk) was purchased from Calbiochem (San Diego, CA). ApoTarget Kit specific to caspase-3 activity assay was obtained from BioSource International, Inc. (San Diego, CA). Ham's F-12, RPMI 1640, penicillin, streptomycin and trypsin/EDTA were obtained from Cellgro (Herndon, VA). The enhanced chemiluminescence western blotting detection reagents were purchased from Amersham Pharmacia Biotech (Piscataway, NJ).

### Cell culture and cell lines

Human non-small cell lung carcinoma cell lines, A549 and H1299, and normal human bronchial epithelial cells were purchased from the American Type Culture Collection (Manassas, VA). The A549 and H1299 cell lines were cultured as monolayers in Ham's F-12 and RPMI 1640 culture medium, respectively, supplemented with 10% heat-inactivated fetal bovine serum (Hyclone, Logan, UT), 100 µg/mL penicillin, and 100 µg/mL streptomycin and maintained in an incubator with a humidified atmosphere of 95% air and 5% CO_2_ at 37°C. The GSPs were dissolved in a small amount of dimethylsulfoxide [DMSO, maximum concentration, 0.1% (v/v)], which was then added to complete cell culture medium prior to addition to subconfluent cells (60–70% confluent). Cells treated with vehicle only (DMSO, 0.1% in media) served as control.

### Analysis of apoptotic cell death by ELISA

Cells were treated with GSPs for 48 h and thereafter harvested. GSPs-induced apoptosis was determined using the Cell Death Detection ELISA Kit (Roche Diagnostics, Palo Alto, CA), which quantifies cytoplasmic histone-associated DNA fragments in the form of mononucleosomes or oligonucleosomes, following the manufacturer's instructions.

### Caspase-3 activity assay

The activity of caspase-3 in cell lysates was measured using the colorimetric protease assay ApoTarget Kit (BioSource International, Inc., CA) following the manufacturer's protocol. Briefly, A549 or H1299 cells were treated with GSPs (20, 40, 60 and 80 µg/mL) for 48 h. Thereafter, the cells were harvested using short trypsinization and cell lysates prepared following the manufacturer's protocol. Samples of the cell lysates (100 µg protein per sample) were mixed with reaction buffer and 200 µmol/L substrate (DEVD-pNA for caspase-3) and incubated for 3 h at 37°C in the dark. The absorbance was then measured at 405 nm and the sample readings calculated by subtracting the absorbance of blank samples.

### DNA cell cycle analysis

Sub-confluent cells (50–60%) were treated with varying concentrations of GSPs in complete medium for 48 h. The cells were then harvested, washed with cold PBS, and processed for cell cycle analysis, as detailed previously [13], [14]. Briefly, the cells (1×10^5^) were re-suspended in 50 µL cold PBS to which 450 µL cold methanol was added and the cells were then incubated for 1 h at 4°C. The cells were centrifuged at 1,100 rpm for 5 minutes; the pellet was washed with cold PBS, re-suspended in 500 µL PBS, and incubated with 5 µL RNase (20 µg/mL final concentration) for 30 minutes. The cells were incubated with propidium iodide (50 µg/mL) on ice for 1 h in the dark. The cell cycle distribution of the cells was then determined using FACSCalibur instrument (BD Biosciences, San Jose, CA) equipped with CellQuest 3.3 software in the Fluorescence-activated Cell Sorting (FACS) machine at the Core Facility of the UAB Comprehensive Cancer Center.

### Immunoprecipitation and western blot analysis

Following treatment of A549 and H1299 cells with GSPs, the cells were harvested, washed with cold PBS and lysed with ice-cold lysis buffer supplemented with protease inhibitors, as detailed previously [12], [13]. For immunoblotting of cytochrome *c*, Smac/DIABLO and Cox IV, mitochondrial and cytosolic fractions were prepared from the cells and the proteins were resolved on 10% Tris-Glycine gels and transferred onto a nitrocellulose membrane. After blocking the non-specific binding sites, the membrane was incubated with the desired primary antibody at 4°C overnight. The membrane was then incubated with appropriate peroxidase-conjugated secondary antibody and the immunoreactive bands were visualized using the enhanced chemiluminescence reagents. Each membrane was stripped and re-probed with anti-β-actin antibody to ensure equal protein loading.

For Cdk inhibitor (Cdki)-Cdk binding assay, A549 cells were treated with vehicle or 60 µg/mL GSPs for 48 h, washed with ice-cold PBS, and whole cell lysates prepared as described previously [13]. Aliquots containing 200 µg of protein were cleared with protein A/G-plus agarose beads (Santa Cruz, CA). Cip1/p21 and Kip1/p27 proteins were immunoprecipitated from whole cell lysates using specific antibodies after incubation for 8 h followed by the addition of protein A/G-plus agarose beads (50 µL, Santa Cruz, CA) and continued incubation overnight at 4°C. Immunoprecipitates were washed, and subsequently subjected to western blot analysis using Tris-Glycine gels followed by immunoblotting using Cdk2, Cdk4 and Cdk6 antibodies.

### Assay for mitochondrial membrane potential

The change in the mitochondrial membrane potential in lung cancer cells after treatment with GSPs was determined by flow cytometry using the fluorescent lipophilic cationic probe JC-1 (5,5′,6,6′-tetrachloro-1,1′,3,3′-tetraethylbenzimidazolcarbocyanine iodide) Detection Kit following the instructions of the manufacturer, and as described by us previously [13], [14]. JC-1 accumulates selectively within intact mitochondria to form multimer J-aggregates emitting fluorescence light at 590 nm. The monomeric form emits light at 527 nm after excitation at 490 nm. Thus, the color of the dye changes from orange to green, depending on the mitochondrial membrane potential, and can be analyzed by FACS with green fluorescence in channel 1 (FL1) and orange emission in channel 2 (FL2).

### Animals and tumor xenograft study

Female athymic nude mice (4–5 weeks-old) were purchased from National Cancer Institute (Frederick, MD), housed in accordance with the Institutional Animal Care and Use Committee guidelines, and provided with sterilized AIN76A diet and water *ad libitum*. All mice were maintained under standard conditions of a 12-h dark/12-h light cycle, a temperature of 24±2°C, and relative humidity of 50±10%. The animal protocol used in this study was approved by the Institutional Animal Care and Use Committee of the University of Alabama at Birmingham, and approved Animal Protocol Number is: 101109267.

To determine the *in vivo* efficacy of GSPs against human lung cancer tumor xenograft growth, exponentially growing A549 and H1299 cells (2×10^6^) were mixed at a 1∶1 ratio with Matrigel (Becton Dickinson, Bedford, MA), and a 100 µL suspension containing 2×10^6^ cells was injected s.c. in the right flank of each mouse. After 24 h, mice were randomly divided in four groups (n = 10). Experimental animals were treated by oral gavage with 50, 100 or 200 mg GSPs/kg body weight/day in 100 µL of PBS five-days a week (Monday through Friday) beginning one day after tumor cell implantation. Control mice received an equal volume of PBS. The experiment was terminated 58 days after tumor cell inoculation. Tumor growth was monitored 2-times per week. Body weight/mouse and diet consumption/mouse were recorded regularly throughout the experiment. Animals were also monitored if they become ill or suffering (lack normal grooming and avoidance behaviors) during the entire experiment period. At the termination of the experiment, the whole tumor mass was harvested, weighed, and a part of the tumor was used for the preparation of tumor lysates to analyze the expression levels of different proteins of interest and remaining part was used for immunohistochemical analysis.

### Immunohistochemical detection of PCNA-positive and TUNEL-positive cells

Five-µm thick tumor sections were deparaffinized and rehydrated in a graded series of alcohols. Following rehydration, an antigen retrieval process was performed by placing the slides in 10 mM sodium citrate buffer, pH 6.0 at 95°C for 20 minutes followed by 20-minutes cooling. The sections were washed in PBS and non-specific binding sites were blocked with 1% BSA with 2% goat serum in PBS prior to incubation with anti-PCNA antibodies for 2 h at room temperature. After washing, the sections were incubated with biotinylated secondary antibody for 45 min followed by HRP-conjugated streptavidin, washing in PBS, incubation with diaminobenzidine substrate, and counterstaining with hematoxylin. Apoptotic cell death in tumor tissues was detected using terminal deoxynucleotidyl transferase-mediated dUTP nick-end labeling (TUNEL) assays. The numbers of PCNA-positive and TUNEL-positive cells were detected and counted using a light microscope. The results are presented as the number of positive cells x 100/total number of cells.

### Statistical analysis

The results from the *in vivo* studies are representative of at least 2–3 independent experiments. The statistical significance of difference between control and GSPs-treated groups were calculated by Student's t test (Sigma Stat 2.03, Jandel Scientific, San Rafael, CA). All quantitative data are shown as mean ± SD. In tumor xenograft study, the statistical significance of difference between control and GSPs-treated groups was determined by ANOVA followed by Bonferroni t test, and in each case P<0.05 was considered statistically significant.

## Results

### NSCLC cells are sensitive to GSPs-induced apoptosis

**Figure 1:**
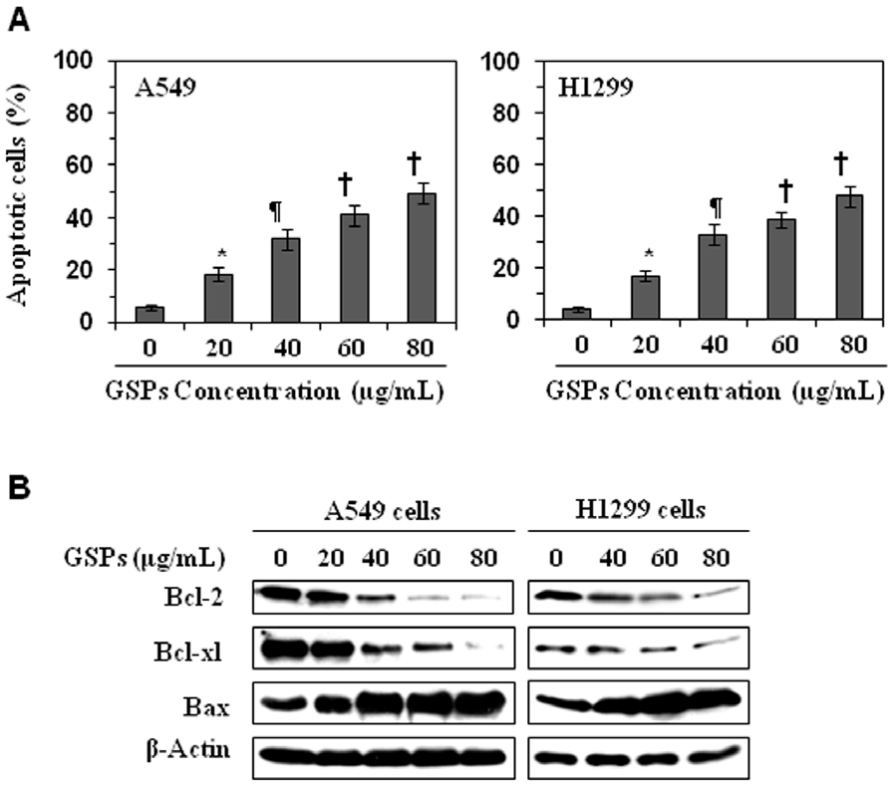
GSPs induce apoptosis in non-small cell lung cancer cells. (**A**) *In vitro* treatment of A549 and H1299 cells with GSPs induces apoptosis in a dose-dependent manner. Apoptotic cell death was analyzed using FACS analysis, and total percentage of apoptotic cells in A549 and H1299 cells are summarized as mean ± SD, n = 3. Significant difference versus non-GSPs-treated controls: ^*^ *P*<0.05; ^¶^ *P*<0.01; ^†^ *P*<0.001. (**B**) Treatment of A549 and H1299 cells with GSPs results in a dose-dependent reduction in the expression of anti-apoptotic proteins, Bcl-2 and Bcl-xl while increasing the expression of the pro-apoptotic protein, Bax, as determined by western blot analysis. Data are representative of three independent experiments with similar results.

We have shown that treatment of A549 and H1299 NSCLC cells with various concentrations of GSPs inhibits the growth or proliferation potential of these cells in a dose- and time-dependent manner. GSPs also induced apoptotic cell death of these cells in a concentration dependent manner [11], [12]. The GSPs-induced apoptosis of A549 and H1299 cells after treatment for 48 h using FACS analysis is summarized in Figure 1A. However, a precise mechanism of anti-apoptotic effects of GSPs against NSCLC cells is not clearly understood. Therefore, the studies were conducted to determine the precise mechanism involved in apoptotic cell death of NSCLC cells by the treatment with GSPs.

### GSPs reduce the expression of the anti-apoptotic proteins Bcl-xl and Bcl-2 while increasing the expression of pro-apoptotic protein Bax in A549 and H1299 cells

The proteins of the Bcl-2 family play a major role in regulation of apoptosis by functioning as promoters (*e.g*., Bax) or inhibitors (Bcl-2 or Bcl-xl) of cell death [15]–[17]. Using western blot analysis, we found that treatment of A549 and H1299 cells with GSPs for 48 h resulted in a dose-dependent reduction in the levels of the anti-apoptotic proteins Bcl-xl and Bcl-2, and an increase in the levels of the pro-apoptotic protein, Bax, as compared with the vehicle-treated control cells (Figure 1 B). Thus, treatment of these lung cancer cells with GSPs can alter the protein levels of key members of the Bcl-2 family in a manner that favors an increase in the ratio of Bax: Bcl-2, which may contribute to the susceptibility of cancer cells to GSP-induced apoptosis [15].

### GSPs induce loss of mitochondrial membrane potential and a subsequent enhancement of the release of cytochrome c and Smac/DIABLO in NSCLC cells

**Figure 2:**
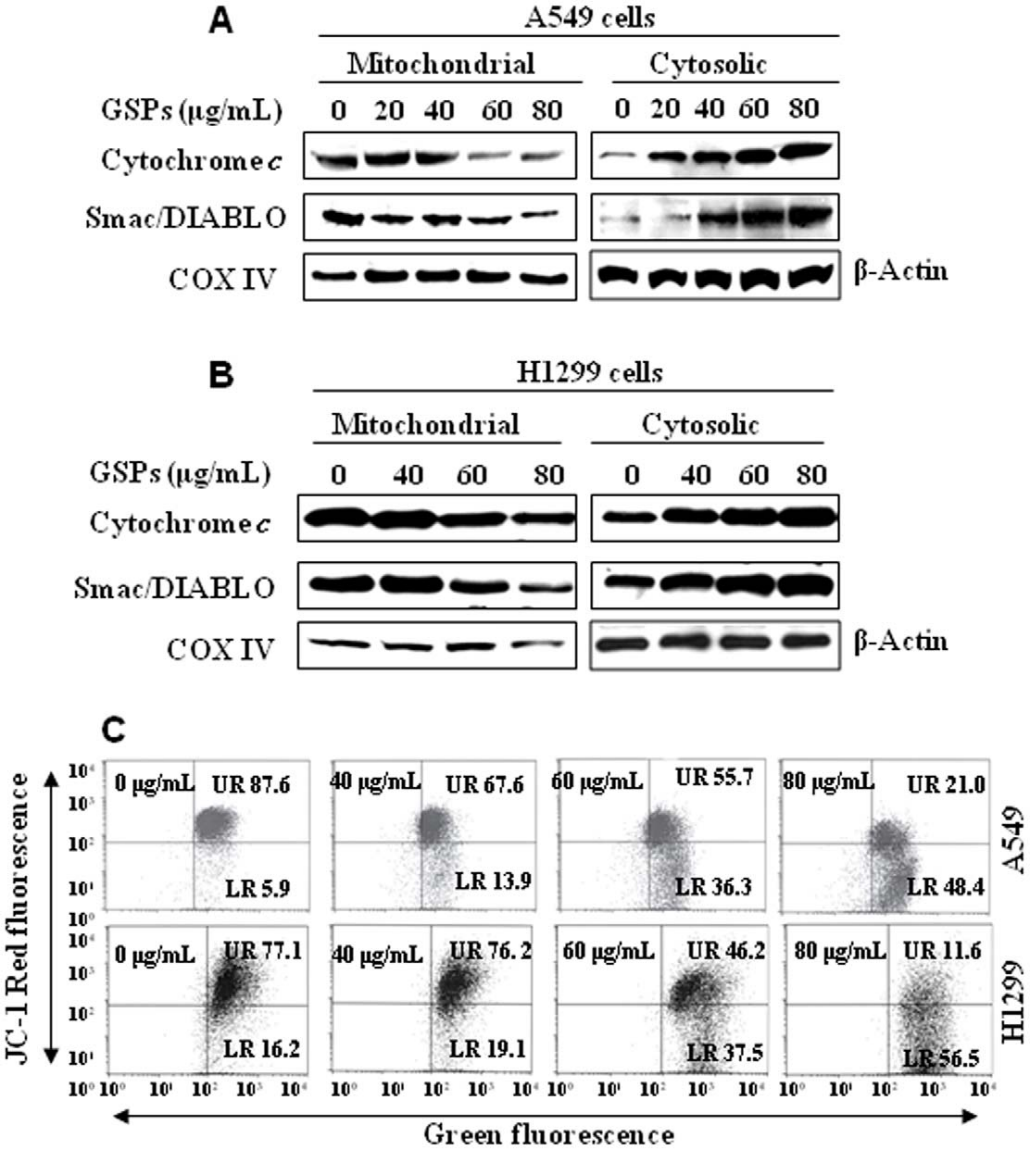
GSPs disrupt mitochondrial membrane potential in non-small cell lung cancer cells (**A & B**) Immunoblotting for cytochrome *c* and Smac/DIABLO using cytosolic and mitochondrial fractions prepared from A549 and H1299 cells following treatment with indicated concentrations of GSPs for 48 h. The blots were stripped and re-probed with anti-COX IV antibody to ensure equal mitochondrial protein loading as well as to rule out cross-contamination of mitochondrial and cytosolic fractions. (**C**) Treatment of A549 and H1299 cells with GSPs induces loss of mitochondrial membrane potential. Cells were treated with indicated doses of GSPs for 48 h. JC-1 dye-stained cell were analyzed by flow cytometry, as described in Materials and Methods. Data are representative of two separate experiments with identical observations.

Loss of mitochondrial membrane potential has been linked to the initiation and activation of apoptotic process in cells [15], [16]. The release of cytochrome c and Smac/DIABLO from mitochondria into the cytosol contributes to the activation of caspases and subsequently leads to apoptotic cell death. To explore the effects of GSPs on this process in the A549 and H1299 cells, cytosolic and mitochondrial fractions were prepared from cells that had been treated with GSPs for 48 h. The results of western blot analysis revealed that treatment of GSPs resulted in a dose-dependent increase in cytochrome c and Smac/DIABLO release into the cytosol and a corresponding reduction in the levels of cytochrome c and Smac/DIABLO in the mitochondrial fractions in both A549 and H1299 cells (Fig. 2A and 2B), thus suggesting the loss of the mitochondrial membrane potential on GSPs treatment in these cells. The blots were stripped and re-probed with anti-COX IV antibody to rule out the possibility of cross-contamination of mitochondrial and cytosolic fractions. The presence of COX IV was not detected in cytosolic fractions.

To further verify that GSPs induce a loss of mitochondrial membrane potential, we used the cationic lipophilic dye, JC-1, which accumulates within mitochondria in a potential-dependent manner. On disruption of the mitochondrial membrane potential, the fluorescence emission of JC-1 dye changes from red to green. Forty-eight h after the addition of the GSPs, the A549 and H1299 cells were harvested, incubated with JC-1 dye and the fluorescence emission analyzed using flow cytometry. As shown in Figure 2C, GSP treatment of A549 and H1299 cells resulted in a dose-dependent increase in the numbers of green-fluorescence-positive cells, as shown in the lower right (LR) quadrant of the FACS histogram. Collectively, these studies suggest that GSPs treatment induce apoptosis of both A549 and H1299 lung carcinoma cells through disruption of the mitochondrial membrane potential.

### GSPs induce the activation of caspases and PARP in both A549 and H1299 cells

**Figure 3:**
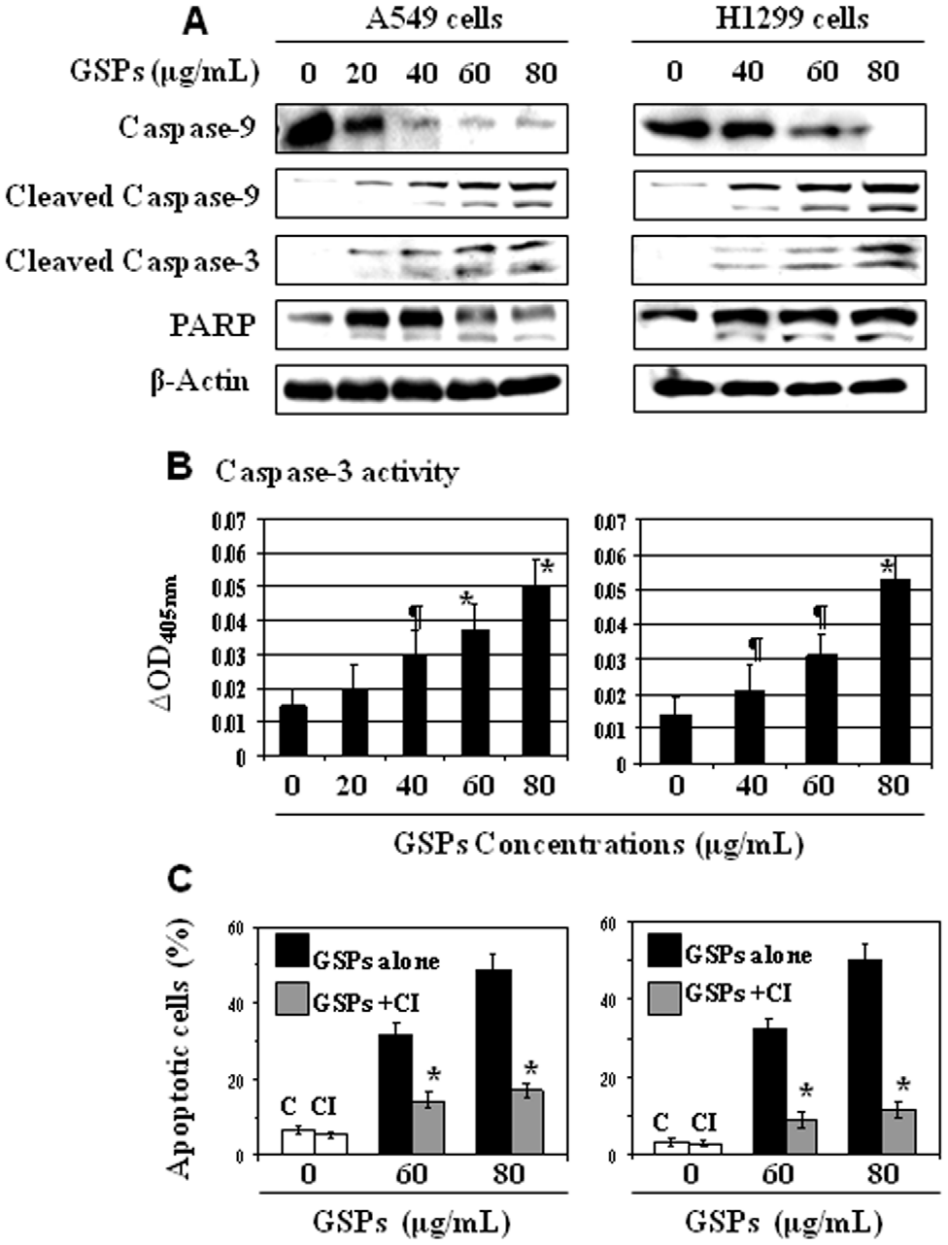
Induction of apoptosis in lung cancer cells by GSPs is mediated through caspase-3 activation (**A**) Treatment of A549 and H1299 cells with GSPs reduces the level of caspase-9 while increases the activation/cleavage of caspase-9, caspase-3 and PARP in a dose-dependent manner. Cells were treated with varying concentrations of GSPs (0, 20, 40, 60 and 80 µg/mL) for 48 h, thereafter cells were harvested, cell lysates prepared and subjected to western blot analysis to detect the levels of caspase-9, cleaved caspase-9, caspase-3 and PARP. A representative blot is shown from three independent experiments with similar results. (**B**) The caspase-3 activity in cell lysates from the samples of Panel A was measured using a colorimetric protease assay (ApoTarget Kit). GSPs treatment to A549 and H1299 cells increases the activity of caspase-3 dose-dependently. Significant difference versus non-GSPs treatment group, ^¶^ *P*<0.01 and ^*^ *P*<0.001. (**C**) The effect of GSPs (60 and 80 µg/mL) on apoptosis of A549 and H1299 cells was determined after 48 h in the absence or presence of 60 µmol/L of the caspase-3 inhibitor (z-DEVD-fmk). The content of apoptotic cells was determined using cell death detection ELISA kit, as detailed in Materials and Methods. The cells treated with z-DEVD-fmk blocked the GSPs-induced apoptosis in A549 and H1299 cells. The percentage of apoptotic cells in different treatment groups was summarized and data are presented as mean ± SD from two repeated experiments. Significant inhibition by caspase-3 inhibitor versus GSPs alone treated cells, ^*^ *P* <0.001. CI = caspase-3 inhibitor, C = control, without any treatment.

The release of the cytochrome *c* and Smac/DIABLO into cytosol activates procaspase 9 in the apoptosome and leads to active caspase-9 cleavage and cleavage of caspase-3 [18]–[20]. Activation of caspase-3 subsequently leads to apoptotic cell death through cleavage of a broad spectrum of target proteins including PARP. Therefore, we determined whether induction of apoptosis of A549 and H1299 cells by GSPs is mediated through the activation of procaspase-9, caspase-3 and PARP proteins. Treatment of A549 and H1299 cells with GSPs for 48 h resulted in a dose-dependent reduction in the levels of caspase-9 while increasing the cleavage of caspases-9, caspase-3 and PARP proteins compared with the vehicle-treated cells (Figure 3A). The GSPs-induced activation of caspase-3 in A549 and H1299 cells also was determined using a colorimetric caspase-3 activity assay. Treatment of both A549 and H1299 lung cancer cells with GSPs for 48 h resulted in a significantly higher (p<0.05; p<0.001) caspase-3 activity in a dose-dependent manner than that observed in the vehicle-treated control cells (Figure 3B).

### Treatment with the caspase-3 inhibitor (z-DEVD-fmk) blocks GSPs-induced apoptosis of both A549 and H1299 cells

To further confirm that GSPs-induced activation of caspase-3 is involved in the induction of apoptotic cell death of A549 and H1299 human lung cancer cells, we determined whether the GSPs-induced apoptosis of A549 and H1299 cells was affected by the addition of the caspase-3-specific inhibitor (z-DEVD-fmk). A549 and H1299 cells had been treated with GSPs with or without z-DEVD-fmk (60 µmol/L) for 48 h. Cells were harvested and cell death was assayed using Cell Death Detection ELISA kit following the instructions of the manufacturer. As shown in Figure 3C (left panel), treatment of A549 cells with GSPs at the concentrations of 60 and 80 µg/mL resulted in 31% and 49% apoptosis or cell death respectively compared to 6.5% in non-GSPs-treated control cells. The treatment of A549 cells with GSPs (60 or 80 µg/mL) in the presence of z-DEVD-fmk resulted in only 14% and 17% apoptosis, respectively, which clearly indicated that the presence of caspase-3-specific inhibitor significantly blocked GSPs-induced apoptotic cells death (*P*<0.001) in A549 cells. Similar results were found when H1299 cells were treated with GSPs in the presence or absence of z-DEVD-fmk, as shown in Figure 3C (right panel). These results indicate that GSPs-induced apoptosis of both A549 and H1299 human lung cancer cells is associated with the activation of caspase-3.

### GSPs induce G1 phase cell cycle arrest in NSCLC cells

**Figure 4:**
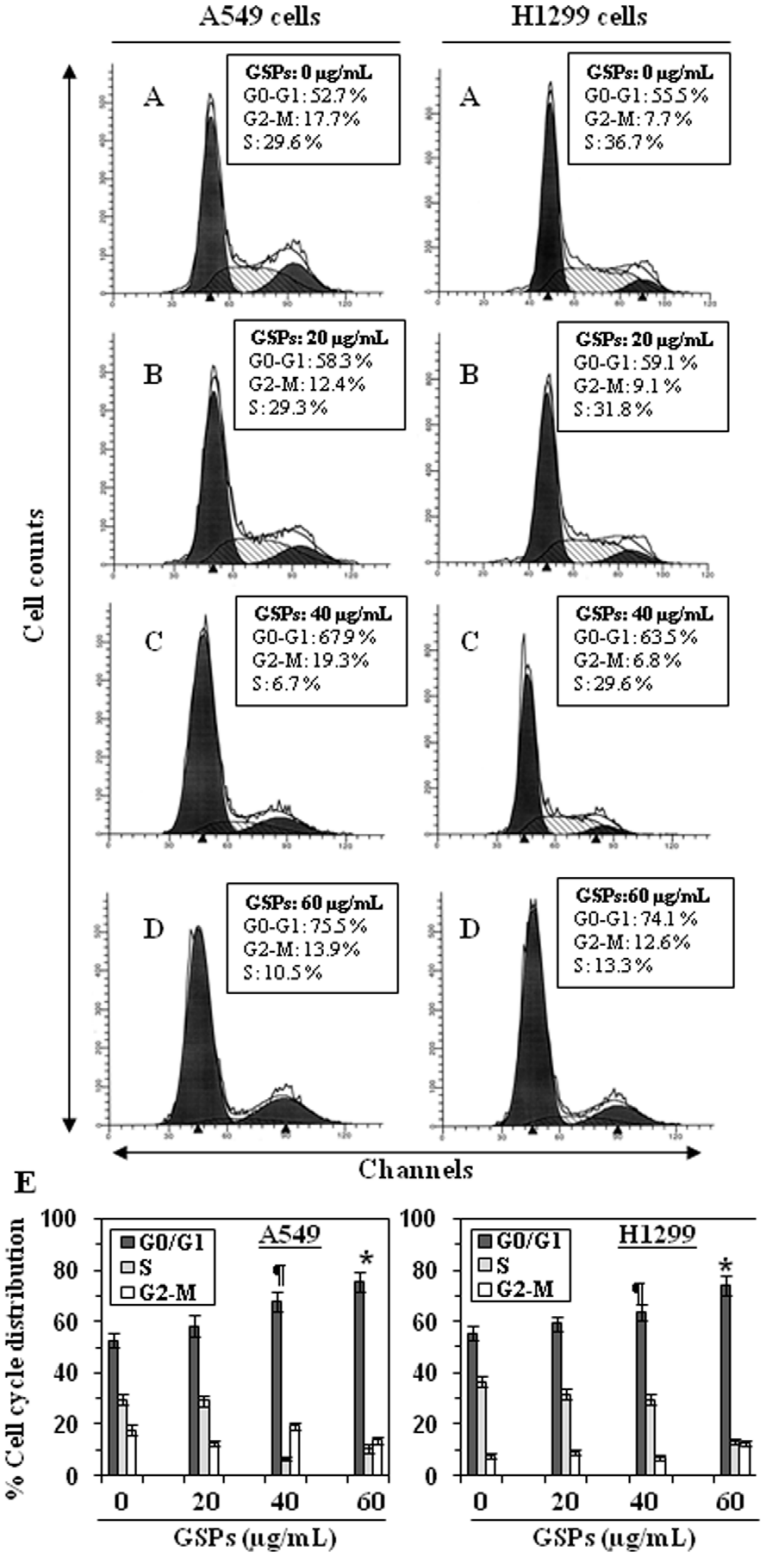
Effect of GSPs on cell cycle progression of A549 and H1299 cells Cells were treated either with vehicle (0.1% DMSO in medium) or 20, 40 and 60 µg/mL doses of GSPs in complete medium. After 48 h of treatment, cells were harvested and digested with RNase. Cellular DNA was stained with propidium iodide and flow cytometric analysis was performed to analyze the cell cycle distribution, as detailed in the Materials and Methods. (**A–D**) Cell cycle distribution in A549 (left panels) and H1299 (right panels) cells with the treatment of various concentrations of GSPs. (**E**) Data from the cell cycle distribution were summarized and presented as the mean ± SD of three independent experiments. Statistically significant versus non-GSPs treated control group, **^¶^** *P* <0.05 and ^*^ *P* <0.01.

Based on the preliminary studies where we observed a strong growth inhibitory effect of GSPs on NSCLC cells, we then determined the possible mechanism of anti-proliferative activity of GSPs which may lead to apoptotic cell death. For this purpose the effect of GSPs on cell cycle progression in A549 and H1299 cells was determined following the treatment with GSPs for 48 h. As summarized in Figure 4, (Panels A–D), treatment of A549 cells with GSPs resulted in a significant higher number of cells in the G1 phase at all the concentrations used: 20 µg/mL (58.3%, *P*<0.05), 40 µg/mL (67.9%, *P*<0.001) and 60 µg/mL (75.5%, *P*<0.001) compared to the non-GSPs treated control (52.7%). As indicated in Figure 4 (Left panels A–D), the dose-dependent effect of GSPs on G1 arrest in A549 cells was largely at the expense of cells in S phase with a minimal change in G2-M cell population compared with the non-GSPs-treated control cells (Panel A). Similar effect of GSPs on G1 phase arrest was also found in H1299 cells (Figure 4, right panels A–D). The results of cell cycle distribution at each dose of GSPs are also summarized in Panel E, which indicated the significant arrest of A549 and H1299 cells in G0–G1 phase after GSPs treatment.

### GSPs decrease the expressions of G1 regulatory proteins of Cdks and cyclins in NSCLC cells

**Figure 5:**
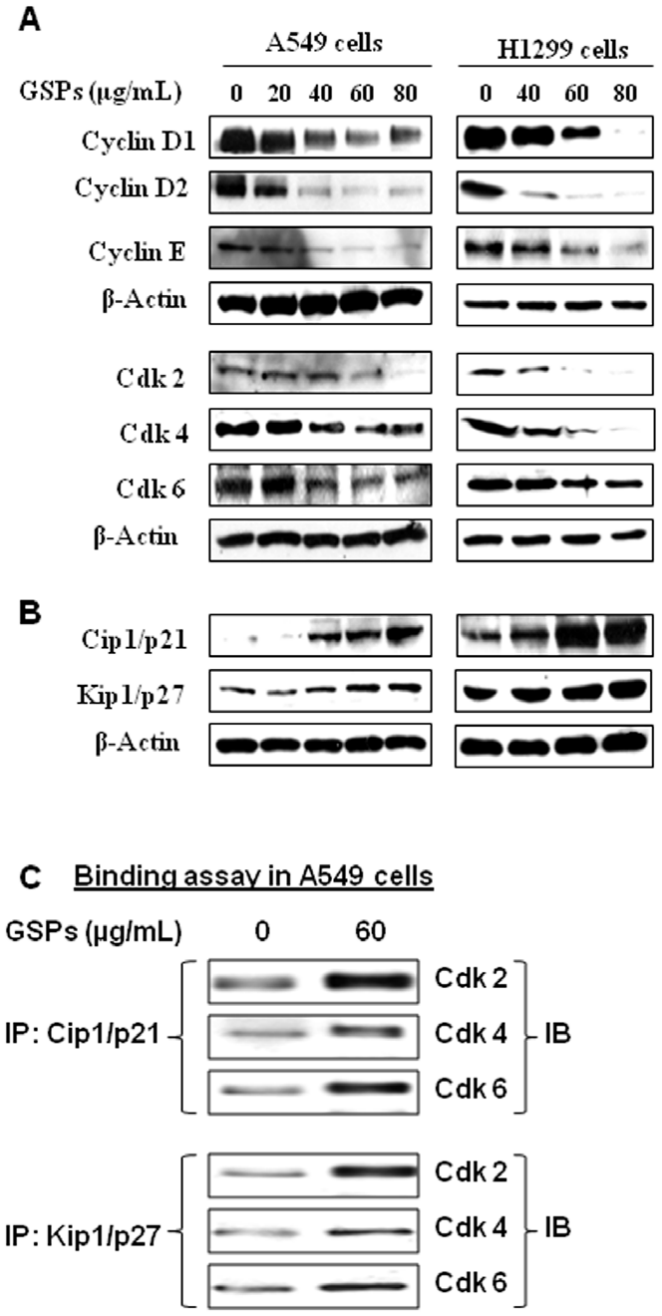
Effect of GSPs on G1 phase cell cycle regulatory proteins in A549 and H1299 cells. The cells were treated with either vehicle (0.1% DMSO in medium) or GSPs (20, 40, 60 and 80 µg/mL) for 48 h and thereafter harvested, cell lysates prepared and then subjected to SDS-PAGE followed by Western blot analysis, as described in Materials and methods. Effect of GSPs was determined on the following: (**A**) the expression of cyclin D1, cyclin D2 and cyclin E, and the expression levels of Cdk2, Cdk4 and Cdk6; and (**B**) the expression of Kip1/p27 and Cip1/p21. β-actin was used to verify equal loading of the samples. Representative blots are shown from three independent experiments with almost identical results. (**C**) In binding assay, Cip1/p21 and Kip1/p27 were immunoprecipitated using specific antibody from total protein lysates followed by SDS-PAGE and western blot analysis for Cdk2, Cdk4 and Ckd6 as detailed in Materials and methods. IP, immunoprecipitation; IB, immunoblotting.

Studies have shown that Cdks and cyclins play crucial roles in the regulation of cell cycle progression [21], therefore we determined the effect of GSPs on the protein levels of the Cdks and cyclins which are negatively regulated by Cdki (Cip1/p21 and Kip1/p27) during G1 phase cell cycle progression. As shown in Figure 5 (Panel A), treatment of A549 cells with GSPs resulted in a marked decrease in the expression of Cdk2, Cdk4 and Cdk6 in a dose-dependent manner at 48 h after GSPs treatment. A strong reduction in Cdks was observed at the doses of 40–80 µg/mL concentrations of GSPs. Similarly, a marked reduction in the expression of cyclins D1, D2 and E was observed in a dose-dependent manner. Identical effect of GSPs was also found when H1299 cells were treated with GSPs and under identical conditions (Figure 5A, right panels).

### GSPs increase the expression of Cdki (Cip1/p21 and Kip1/p27) in NSCLC cells

The Cdki regulate the progression of cells in the Go/G1 phase of the cell cycle and induction of Cip1/p21 and Kip1/p27 causes a blockade of the G1 to S transition, thereby resulting in a Go/G1 phase arrest of the cell cycle [22]. The loss of Cdki in human cancers leads to uncontrolled cell proliferation [23]. In this context, our results revealed that treatment of human NSCLC cells, A549 and H1299, with varying concentrations of GSPs (0, 20, 40, 60 and 80 µg/mL) for 48 h resulted in a dose-dependent increase in protein expression of Kip1/p27 and Cip1/p21 (Figure 5 B). The GSPs-induced increase in inhibitory Cdki proteins may have a role in blockade of A549 and H1299 cells in G1 phase. These changes in protein expression were not due to differences in the amount of proteins loaded on the gels as the equivalent protein loading was confirmed by probing stripped blots for β-actin, as shown in Figure 5B.

### GSPs increase Cdk-Cdki binding in NSCLC cells

As GSPs induce the expression of Cdki in both A549 and H1299 cells, and since the induction of the Cdki has been shown to result in an increased interaction with Cdks leading to a decrease in their kinase activity [24], we next determined whether GSPs promote the interaction between Cdki and Cdk. To assess this effect, Cip1/p21 and Kip1/p27 were immunoprecipitated from total cell lysates of A549 and their binding with Cdk2, Cdk4 and Cdk6 was assessed using western blot analysis. As compared to vehicle treated controls, treatment with GSPs was found to enhance the binding of Cdk2, Cdk4 and Cdk6 with Cip1/p21 and Kip1/p27 (Figure 5C). These results suggest that an increased interaction between Cdki with Cdks plays an important regulatory role in the GSPs-induced G1 arrest of cell cycle progression in human non-small cell lung cancer cells, possibly through the decrease in their Cdk kinase activity.

### Administration of GSPs by oral gavage inhibits the growth of tumor xenografts: GSPs are non-toxic to mice

**Figure 6:**
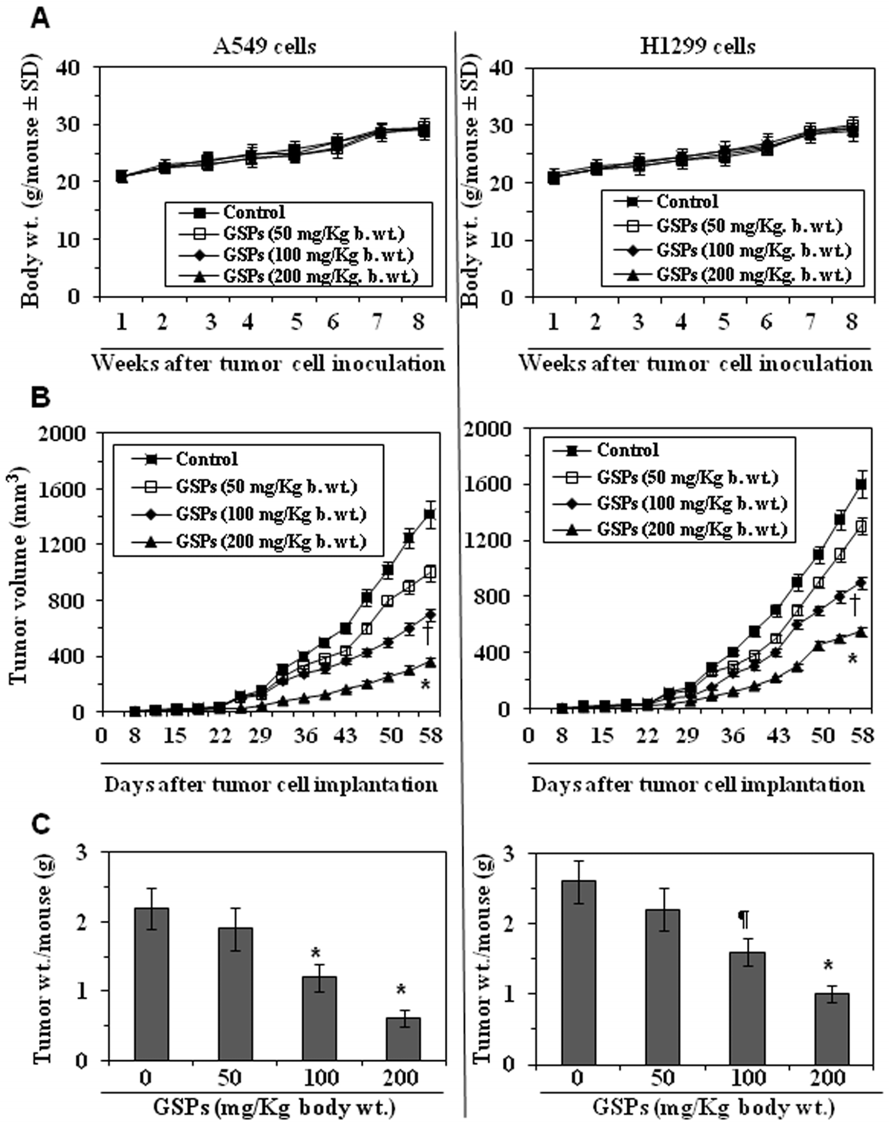
Administration of GSPs by gavage inhibits the growth of lung tumor xenografts in mice. Each mouse was *s.c.* implanted with either 2×10^6^ A549 or H1299 cells mixed with Matrigel on the right flank. Twenty-four h later, mice were given either PBS (100 µL) or GSPs (50, 100 or 200 mg/kg body weight in 100 µL PBS) by gavage 5 days/week. (**A**) Change in body weight of mice during the 8 weeks study was monitored. The body weights of the control and GSPs-fed mice did not differ significantly throughout the experiment protocol. (**B**) Average tumor volume ±SD/mouse (mm^3^) in each group. (**C**) Tumors were harvested at the termination of the experiment, and the wet weight of the tumor/mouse in grams in each group is reported as mean ± SD. Statistical significance of difference between control and GSPs-fed groups was analyzed by one-way ANOVA followed by Bonferroni *t* test. n = 10. Statistical significance *vs* non-GSPs-fed controls, ^¶^ *P*<0.05; ^†^ *P*<0.01; ^*^ *P*<0.005.

Next, we determined the effect of GSPs on A549 and H1299 xenografts growth in immuno-compromised athymic nude mice. Three different doses of GSPs, *i.e*., 50, 100 and 200 mg GSPs/kg body weight of mice were used and the effects on the growth of the tumors were monitored. The body weights and consumption of diet and water per animal in each treatment group was recorded on weekly basis. The average body weight of the GSPs-treated and non-GSPs-treated control mice remained identical throughout the duration of the experiment protocol (Figure 6A). The daily consumption of diet or drinking water by the mice in each group was also almost identical (data not shown) and the mice that were treated with GSPs did not exhibit impaired movement and posture or any other sign of physical toxicity. These data suggest that administration of GSPs by oral gavage at the concentrations used in these studies is not associated with visible toxicity in laboratory animals.

Periodic measurement of the tumor xenograft volume indicated that the treatment with GSPs, particularly with the doses of 100 and 200 mg GSPs/kg body weight, resulted in reduced growth of both the A549 and H1299 lung cancer xenografts throughout the duration of the experiment. As can be seen in Figure 6B (left panel), the GSPs-induced inhibition of A549 xenograft tumor volume was 51% in mice administered GSPs at a concentration of 100 mg/kg body weight and 75% (*P*<0.001) in the mice administered GSPs at a concentration of 200 mg/kg body weight. Significant inhibition of the H1299 xenograft tumors also was observed after the treatment of mice with GSPs (Figure 6B, right panel).

At the termination of the experiment on the 58^th^ day of the experiment, the wet weight of the tumor/mouse in each treatment group was recorded. As shown in Figure 6C (left panel), the wet weight of the A459 xenograft tumor/mouse was significantly lower in the mice administered GSPs than in the mice that did not receive GSPs. The wet weight of the A549 tumors was 14% lower in mice administered 50 mg GSPs/kg body weight; 45% lower (*P*<0.001) in mice administered 100 mg GSPs/kg body weight; and 71% lower (*P*<0.001) in mice administered in 200 mg GSPs/kg body weight compared with non-GSPs-treated control mice. Administration of GSPs at the same concentrations also resulted in a reduction in the wet weight of the H1299 tumor xenografts (Figure 6C, right panel). The wet weight of the H1299 tumors was 15% lower (not significant) in mice administered 50 mg GSPs/kg body weight; 39% lower (*P*<0.01) in mice administered 100 mg GSPs/kg body weight; and 62% lower (*P*<0.001) in mice administered in 200 mg GSPs/kg body weight.

### GSPs inhibit the growth of A549 and H1299 tumor xenografts through the enhancement of apoptotic cell death of tumor cells

**Figure 7:**
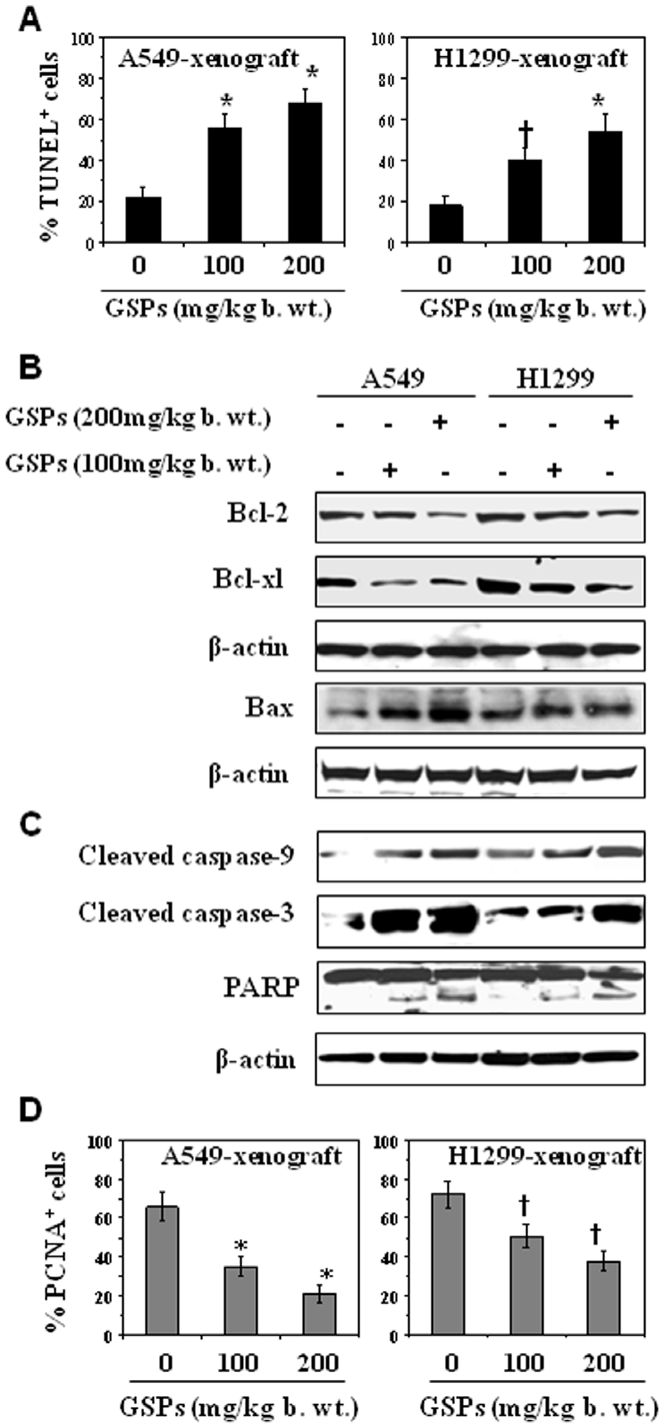
Administration of GSPs by gavage induces apoptosis in tumor xenograft cells. (**A**) The data on terminal deoxynucleotidyl transferase-mediated nick-end labeling (TUNEL)-positive cells in tumor xenograft tissues from GSPs-treated and GSPs-untreated samples are summarized. (**B**) GSPs inhibit the expression of anti-apoptotic proteins while increases the expression of pro-apoptotic protein in A549 and H1299 xenograft tissues. (**C**) GSPs enhance the activation of caspase-9, caspase-3 and PARP proteins in tumor xenografts. Tumor lysates were prepared from the tumors collected at the termination of the experiment and subjected to western blot analysis, as described in Materials and methods. Representative blots are presented from the independent experiments from at least six tumors from six different mice per group with identical observations. (**D**) Analysis of PCNA-positive cells for proliferation index. Immunohistochemical data in terms of percentage of positive cells (TUNEL-positive or PCNA-positive) are summarized and presented as mean ±SD of 6–7 tumor samples from each group. Statistical significance versus non-GSPs-fed controls, ^†^ *P*<0.01; ^*^ *P*<0.001.

To determine if administration of GSPs by gavage inhibits the growth of tumor xenografts by enhancing the apoptosis of the lung tumor cells *in vivo*, the xenograft tumors were subjected to TUNEL-positive staining. As shown in Figure 7A, the percentages of TUNEL-positive cells were significantly higher (*P*<0.001) in the A549 and H1299 xenograft tumors of the GSPs-treated mice as compared to percentages of TUNEL-positive cells in the xenograft tumors in the control mice that did not receive GSPs. The percentage of TUNEL-positive cells was comparatively higher in the A549 xenograft tumors than the H1299 xenograft tumors.

To further determine the mechanisms involved in the GSPs-mediated induction of apoptosis *in vivo* tumor cells, we examined tumor xenograft samples from each of the treatment groups for the expressions of pro- and anti-apoptotic proteins of the Bcl-2 family using western blot analysis. As shown in Figure 7B, the levels of the anti-apoptotic proteins, Bcl-2 and Bcl-xl, were lower in the A459 and H1299 xenograft tumors from mice treated with GSPs than in the tumors in control mice that did not receive GSPs, whereas the levels of the pro-apoptotic protein, Bax, were higher. The cleavage of caspase-3 and caspase-9 proteins was markedly higher in the tumor from the GSPs-treated mice than the control mice. Similarly, the cleavage of PARP protein was enhanced, as shown in Figure 7C.

Additionally, we determined whether GSPs treatment also affects the tumor cell proliferation potential in the A549 and H1299 tumor xenografts. As summarized in Figure 7D, immunohistochemical detection analysis of PCNA-positive cells in tumor xenograft tissues indicated that the percentage of proliferating cells was significantly lower in both the A549 (*P*<0.001) and H1299 (*P*<0.01) tumor xenografts from GSPs-treated mice than the xenograft tumors from the control mice.

### Administration of GSPs decreases the expressions of G1 regulatory proteins of Cdks and cyclins while increases the levels of Cdk inhibitory proteins in tumor xenograft tissues of NSCLC cells

**Figure 8:**
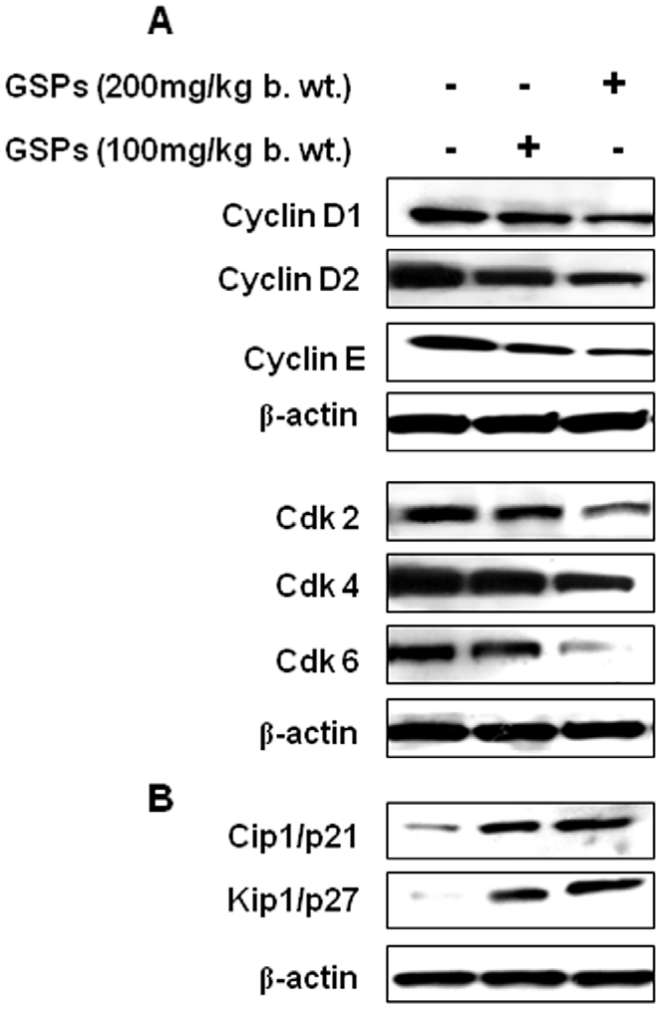
Effect of dietary GSPs on G1 phase cell cycle regulatory proteins in xenograft tissues. The A549 tumor xenograft tissues were harvested at the termination of the experiment, and tumor lysates were subjected to the analyses of cell cycle proteins of G1 phase using Western blot analysis, as described in Materials and Methods. Effect of GSPs was determined on the following: (**A**) the expression of cyclin D1, cyclin D2 and cyclin E, and the expression levels of Cdk2, Cdk4 and Cdk6; and (**B**) the expression of Cdk inhibitory proteins, Kip1/p27 and Cip1/p21. β-actin was used to verify equal loading of the samples. Representative blots are presented from the independent experiments from at least six tumors from six different mice per group with identical expression levels of proteins.

As the inhibitory effect of GSPs on the tumor xenograft growth of A549 and H1299 cells was identical, we selected the tumor tissues of A549 cells for further analyses of cell cycle regulatory proteins. As shown in Figure 8A, treatment of mice with GSPs resulted in a marked decrease in the expression of Cdk2, Cdk4 and Cdk6 compared to the tumors from non-GSPs-treated mice in a dose-dependent manner. Similarly, a marked reduction in the expression of cyclins D1, D2 and E was also observed.

As the Cdki regulate the progression of cells in the Go/G1 phase of the cell cycle, we also examined the effect of GSPs on the levels of Cdk inhibitory proteins in tumor xenograft tissues from each treatment group. Western blot analysis revealed that administration of GSPs resulted in a dose-dependent increase in protein expression of Kip1/p27 and Cip1/p21 (Figure 8B). The GSPs-induced increase in the levels of Cdki proteins may have a role in controlling the cell cycle progression and that may have resulted in suppression of tumor growth.

## Discussion

Chemotherapy offers a promising strategy for control of cancer. Several phytochemicals including dietary plant products have been shown to have anti-carcinogenic properties. GSPs represent one such phytochemicals and have been shown to have anti-carcinogenic activity in different organs [10]–[12], [25], [26]. We have shown previously that long-term dietary feeding of GSPs supplemented with AIN76A control diet of laboratory animals does not show apparent signs of toxicity [10], [11]. In the present study, we further verify that long-term administration of GSPs by oral gavage also does not induce any visible sign of toxicity in mice. Similarly, *in vitro* studies indicate that GSPs exhibit anti-proliferation or cytotoxic effects against tumor cells, but do not exhibit toxic effects on the growth and viability of normal (non-neoplastic) cells in culture [11], [12], [25]. Previously we have shown that treatment of both A549 and H1299 human non-small cell lung cancer cells with GSPs resulted in inhibition of cell proliferation and induction of apoptosis, while these effects of GSPs were not observed in normal human bronchial epithelial cells [11], [12]. Induction of apoptosis is considered as one of the possible mechanisms of inhibition of cancer development, and many therapeutic agents have been shown to act through the induction of apoptosis in their inhibition or blockade of the carcinogenic process [27]–[29]. Some evidence indicates that apoptosis may represent a protective mechanism against neoplastic development by eliminating genetically damaged cells or those cells that have improperly been induced to proliferate by factors such as carcinogens [30], [31].

Experimental evidence suggests that apoptosis can be mediated by several different pathways and that there are numerous regulatory molecules associated with these pathways. The proteins of the Bcl-2 family include both pro- and anti-apoptotic members that elicit opposing effects on mitochondria [15], [32], [33]. Enhancement of pro-apoptotic Bax over Bcl-2 proteins can enhance the permeability of the mitochondrial membrane, which in turn results in the release of apoptogenic factors. Repression of anti-apoptotic members of this family, including Bcl-2 and Bcl-xl, preserves the integrity of the mitochondria. This blocks the release of soluble inter-membrane factors such as cytochrome *c* that activate the effectors of apoptosis [34]. In this study, we found that treatment of the NSCLC cells with GSPs up-regulate the pro-apoptotic Bax protein and down-regulate the anti-apoptotic proteins Bcl-2 and Bcl-xl and that this occurs in both A549 and H1299 cells (Figure 1). The alteration in the Bax/Bcl-2 ratio is critical for apoptosis and causes the release of cytochrome c and Smac/DIABLO from mitochondria into the cytosol after the loss of mitochondrial membrane potential [15], [16]. Cytosolic cytochrome c can bind to Apaf-1 and activate caspase-9 in the apoptosomes in response to diverse inducers of cell death [17], [35]. Activation of caspase-9 leads to the activation of caspase-3 which is one of the key-mediators of apoptosis [15], [35]. GSPs also result in an increase in the release of cytochrome c and Smac/DIABLO in cytosols (Figure 2), activation of caspase-9 and finally an increase in the activation or cleavage of caspase-3 and PARP (Figure 3). The involvement of GSPs-induced increase in activation of caspase-3 and its effect on apoptosis was further confirmed by measuring its activity and induction of apoptotic cell death by cell death detection ELISA. The blockade of GSPs-induced apoptosis in A549 and H1299 cells by the addition of the caspase-3 inhibitor (z-DEVD-fmk) confirmed the role of activated caspase-3 in the GSPs-induced apoptosis.

It has been recognized that control of cell cycle progression in cancer cells is an effective strategy to inhibit tumor growth [22], [36] as the molecular analyses of human cancers have revealed that cell cycle regulators are frequently deregulated in most of the common malignancies [37], [38]. Our *in vitro* data demonstrate that treatment of A549 and H1299 cells with GSPs induces G1 phase arrest of cell cycle progression (Figure 4) indicating that one of the mechanisms by which GSPs inhibit the proliferation of lung cancer cells is inhibition of cell cycle progression. Our studies demonstrate a marked decrease in the expressions of cyclins D1, D2 and E, and Cdk2, Cdk4 and Cdk6 in both A549 and H1299 cells dose-dependently on GSPs treatment suggests the disruption of the uncontrolled cell cycle progression of human NSCLC cells (Figure 5) and that the GSPs-induced G1 arrest is mediated through the up-regulation of cyclin-dependent kinase inhibitory proteins, Cip1/p21 and Kip1/p27, which enhances the formation of heterotrimeric complexes with the G1/S Cdks and cyclins thereby resulting in inhibition of their activity.

Uncontrolled cell division depends on the activation of cyclins, which bind to Cdk to induce cell cycle progression towards S phase. Cdk kinase activity is one of the major causes of cancer progression, and their functions are tightly regulated by Cdki, such as, Cip1/p21 and Kip1/p27 proteins. Cip1/p21 is a universal inhibitor of Cdk(s) and Kip1/p27 is commonly up-regulated in response to anti-proliferative signals [39]. The increased expression of G1 cyclins in cancer cells provides them an uncontrolled growth advantage because most of these cells either lack Cdki, harbor nonfunctional Cdki, or Cdki expression is not at a sufficient level to control Cdk-cyclin activity [38], [40]. Consistent with these reports, the increased expressions of Cdkis together with decreased expression of cyclins and Cdks on the GSPs treatment to A549 and H1299 cells suggest that GSPs might be effective as a chemotherapeutic agent for the treatment of non-small cell lung cancers.

Our *in vivo* study provides further evidence that administration of GSPs by gavage inhibits the growth of both A549 and H1299 tumor xenografts by inducing cell death and without any apparent sign of toxicity in athymic nude mice (Figures 6 and 7). Further, consistent with the findings in cell culture model, an increase in the levels of pro-apoptotic protein Bax, and cleaved caspase-9, caspase-3 and PARP proteins were also observed in the tumors of GSPs-treated group compared to the control mice which were not given GSPs by gavage (Figure 7). These results also indicate the inhibition of growth of lung tumor xenograft through increased apoptosis of tumor cells and inhibition of tumor cell proliferation in GSPs-treated mice.

It is reasonable to consider whether the chemotherapeutic effect of GSPs examined in an animal model can be translated in human system. For appropriate conversion of the doses of chemopreventive or chemotherapeutic agent from animal studies to humans, the body surface area normalization method has been recommended [41]. In the present study, maximum dose of GSPs by oral gavage was given 200 mg/kg body weight of the mouse. Based on this information, the human equivalent dose of GSPs was calculated using the following formula:



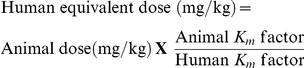



(*Km* factor for mouse = 3; *Km* factor for adult human = 37).

If, the body weight of a normal standard person is considered to be 70 Kg, then 1.13 g GSPs will be required for a healthy person/day to produce same level of anti-lung carcinogenic effects as observed in mice, which seems reasonable, affordable and attainable.

In summary, the novelty of this study lies in the analysis of chemotherapeutic effects of GSPs on additional new molecular targets of NSCLC cells using both *in vitro* and *in vivo* models. This detailed and systematic study revealed that GSPs induce apoptosis of human non-small cell lung cancer cells by loss of mitochondrial membrane potential, which was not reported earlier in this model. The present findings provide pre-clinical data suggesting that grape seed proanthocyanidins have the potential to be developed as a pharmacologically safe agent either alone or in combination with other drugs for the treatment of non-small cell lung cancers in humans.
