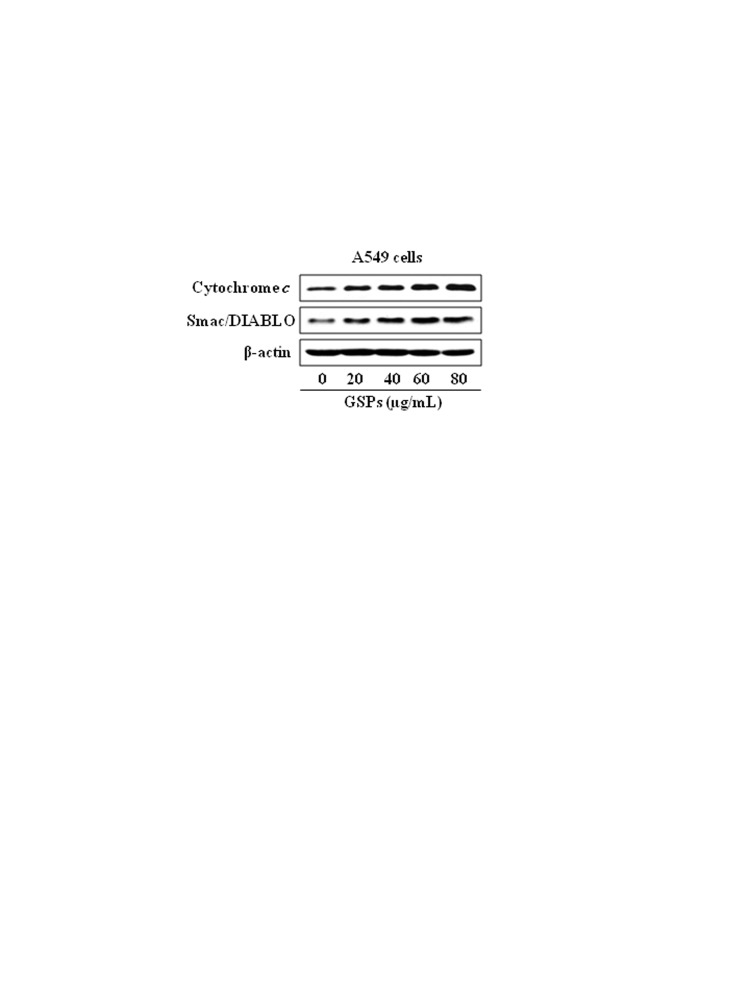# Correction: Grape Proanthocyanidins Induce Apoptosis by Loss of Mitochondrial Membrane Potential of Human Non-Small Cell Lung Cancer Cells *In Vitro* and *In Vivo*

**DOI:** 10.1371/annotation/23a8e553-4fce-4c73-9946-7a2a6a5729e9

**Published:** 2012-06-07

**Authors:** Tripti Singh, Som D. Sharma, Santosh K. Katiyar

In the published article, the beta-actin blot under Figure 2A (cytosolic) was duplicated unknowingly by error with other published papers (Figure 6B; Sharma et al., Mol. Cancer Ther, 9(3); 569, 2010; and Fig. 2B; Sharma and Katiyar, Pharm Res., 27: 1092, 2010). The authors would like to apologize to readers and to the editors for this error.

The experiments were repeated in A549 cells with and without treatment with grape seed proanthocyanidins (GSPs) under identical conditions. Cytosolic fractions were subjected to Western blot analysis, and new data was generated to confirm and verify equal protein loading on the gel using antibody against β-actin and to replace the duplicated one. The data obtained confirm the results originally reported in the article. The corrected figure is available here: 

**Figure:**